# Kinetics and Mechanism of the Reaction between Chromium(III) and 2,3-Dihydroxybenzoic Acid in Weak Acidic Aqueous Solutions

**DOI:** 10.1155/2010/348692

**Published:** 2010-05-31

**Authors:** Athinoula L. Petrou, Vladimiros Thoma, Konstantinos Tampouris

**Affiliations:** Laboratory of Inorganic Chemistry, University of Athens, Panepistimioupolis, 15771 Athens, Greece

## Abstract

The reaction between chromium(III) and 2,3-dihydroxybenzoic acid (2,3-DHBA) takes place in at least three stages, involving various intermediates. The ligand (2,3-DHBA)-to-chromium(III) ratio in the final product of the reaction is 1 : 1. The first stage is suggested to be the reaction of [Cr(H_2_O)_5_(OH)]^2+^ with the ligand in weak acidic aqueous solutions that follows an *I*_*d*_ mechanism. The second and third stages do not depend on the concentrations of chromium(III), and their activation parameters are Δ*H* ^≠^_2(obs)_ = 61.2 ± 3.1 kJmol^−1^, Δ*S* ^≠^_2(obs)_ = −91.1 ± 11.0 JK^−1^mol^−1^, Δ*H* ^≠^_3(obs)_ = 124.5 ± 8.7 kJmol^−1^, and Δ*S* ^≠^_3(obs)_ = 95.1 ± 29.0 JK^−1^mol^−1^. These two stages are proposed to proceed via associative mechanisms. The positive value of Δ*S* ^≠^_3(obs)_ can be explained by the opening of a four-membered ring (positive entropy change) and the breaking of a hydrogen bond (positive entropy change) at the associative step of the replacement of the carboxyl group by the hydroxyl group at the chromium(III) center (negative entropy change in associative mechanisms). The reactions are accompanied by proton release, as shown by the pH decrease.

## 1. Introduction

Pathogens have developed many strategies to cope with iron limitation caused by plants and mammals in order to restrict their unwanted growth in them. One of the best-known methods (strategies) is through the synthesis of small molecules that can act as iron chelators known as siderophores [1]. 2,3-Dihydroxybenzoic acid (2,3-DHBA) is a monocatechol siderophore. This ligand is a triprotic acid H_3_L, and the values of *K*_1_ and *K*_2_ refer to the protonation constants of the two hydroxylate groups and *K*_3_ refers to that of the carboxyl group. At 25°C and Ionic Strength 0.2 M the average values of the accepted constants for 2,3-DHPA are log(*K*_2_/M^−1^) = 9.86 ± 0.04 and log(*K*_3_/M^−1^) = 3.00 ± 0.27. log(*K*_1_/M^−1^) ranges between 10 and >14 at 25°C and Ionic Strength 0.02–1.0 M [2]. In the molecule, intramolecular hydrogen bonds are formed between the hydroxyl groups and the carboxyl group [3]. Complexes of 2,3-DHBA with various metal ions, for example, Al^3+^, VO^2+^, Mn^2+^, Fe^3+^, Cu^2+^, Cd^2+^, and so forth, have been prepared and their equilibrium constants have been determined [2]. Catecholic type of coordination was also suggested for the Fe(III)-2,3-DHBA complex [4, 5]. 

Chromium(III) is an essential for life trace element and its role has been extensively studied. At low concentrations it is beneficial to many plant species, whereas, in the same plants, at higher concentrations it is toxic. In the roots, concentrations of 175 ppm have been shown to be harmless. At concentrations between 375 and 400 ppm toxicities were evident. The above suggests the existence of a tolerating mechanism in the roots [6].

**Figure 1:**
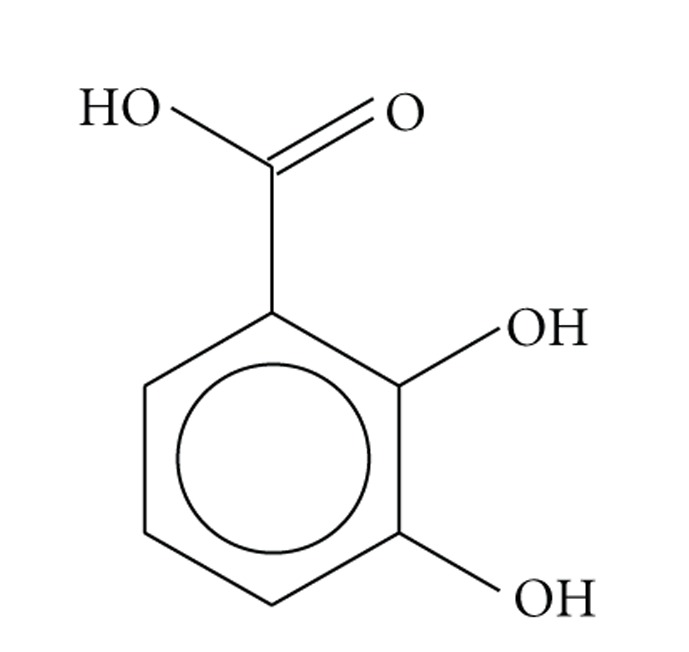
2,3-Dihydroxybenzoic acid (2,3-DHBA).

 The aim of this work is to study and determine the kinetics and suggest a mechanism of the substitution reactions between chromium(III) and 2,3-DHBA. Knowledge of the mechanism will further contribute in the understanding of the role of organic ligands in the uptake of metals by plants because metal ions are transferred by organic ligands from the roots to certain parts of the plants. Such ligands are humic substances and products of their decomposition, that is, organic acids. One of such organic ligands is 2,3-DHBA (Figure 1).

## 2. Experimental Section

### 2.1. Reagents and Materials

The reactants used were of analytical grade. 2,3-DHBA (Ferak Berlin) was used as received and was dissolved in dilute (0.1 M) solution of KOH (Merck) for pH adjustment for the ligand to be dissolved, in concentrations ranging from 3.9 × 10^−3^ to 7.8 × 10^−3^ M. Stock solutions of Cr(III) were prepared from Cr(NO_3_)_3_ · 9H_2_O (Fluka). The chromium(III) concentrations ranged between (5–14) × 10^−2^ M. The ionic strength was adjusted using KNO_3_. The solutions of 2,3-DHBA were used soon after their preparation in order to avoid transformation and decomposition reactions. The addition of the Cr(III) solution kept the pH below 4 due to its acidic hydrolysis:

(1) $$\text{Cr}{\left({\text{H}}_{2}\text{O}\right)}_{6}^{3+}⇌\text{Cr}{\left({\text{H}}_{2}\text{O}\right)}_{5}{\left(\text{OH}\right)}^{2+}+{\text{H}}^{+}$$

**Figure 2:**
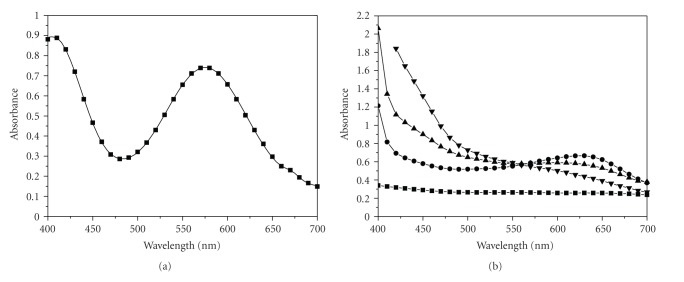
UV-Vis spectra of Cr(III) (a) and 2,3-DHBA (b). Conditions: (a) Chromium(III) Spectrum: [Cr(III)] = 0.050 M, *T*=298 K. Spectrophotometric cell path *d* = 1 cm. (b) 2,3-DHBA Spectra: [2,3-DHBA] = 0.040 M, *T* = 298 K. –■– 2,3-DHBA reduced, –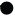
– 2,3-DHBA oxidized (minutes), –▲– 2,3-DHBA oxidized (hours), –▾– 2,3-DHBA oxidized (a few days). Spectrophotometric cell path *d* = 1 cm.

The UV-Vis spectrum of the chromium(III) solution and of the ligand along with spectra of its oxidation products at various times after dissolution are given in Figure 2. 

### 2.2. Kinetic Experiments

**Figure 3:**
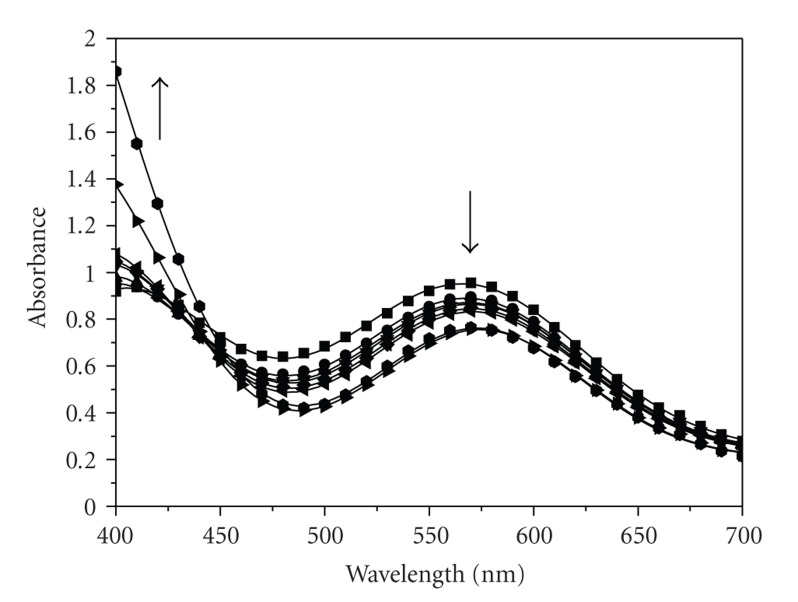
UV-Vis spectra of Cr(III)/2,3-DHBA mixture at various times after mixing. Conditions: [Cr(III)]_0_ = 0.036 M, [2,3-DHBA]_0_ =  0.011 M, and *T* = 296 K. Spectrophotometric cell path *d* = 1 cm. –■– 0:00 hours –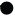
– 1:00 hour, –▲– 2:00 hours, –▾– 3:00 hours, –♦– 4:00 hours, –◂– 5:00 hours, –▸– 29:00 hours, –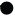
– a few days.

All kinetic experiments were conducted at pH values below 4 in the presence of air. The kinetics was followed spectrophotometrically by recording the absorbances at various reaction times after mixing (Figure 3). The absorbances were recorded on a Hitachi Model 100-60 spectrophotometer. They refer to the substitution of water molecules in the [Cr(H_2_O)_6_]^3+^ coordination sphere by 2,3-DHBA and were followed at 573 nm where the biggest absorbance difference between the final product and the initial mixture of [Cr(H_2_O)_6_]^3+^/2,3-DHBA exists.

First-order rate constants were estimated with a nonlinear least-squares fit.

Pseudo-first-order conditions, that is, excess of Cr(III) which results in acidic solution due to its hydrolysis, were applied for all the kinetic experiments. Experiments in excess of ligand (2,3-DHBA) were not possible to be performed due to low solubility and oxidation problems of the ligand in alkaline solutions in air. The alkaline solution results in the reaction mixture under the experimental conditions. The KOH solution is added in order to enhance solubility. The oxidation of the ligand is shown in Figure 2, lower spectra. 

The temperatures of the kinetic experiments ranged between 16°C and 37°C to avoid acceleration of the ligand's decomposition/autoxidation.

**Figure 4:**
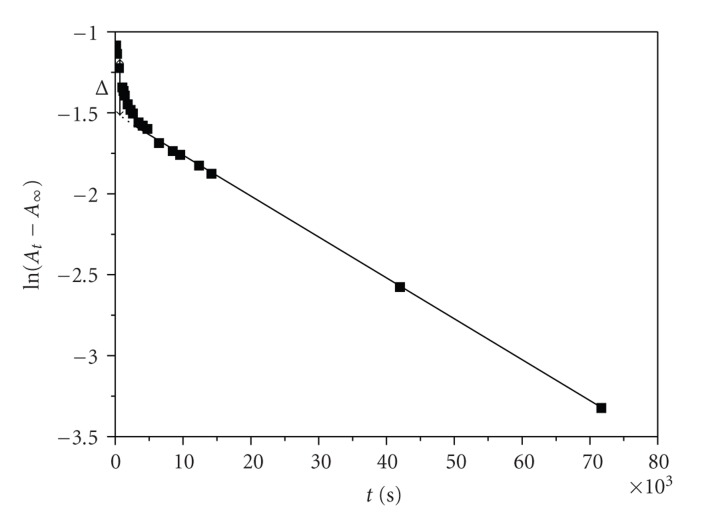
A typical non-linear plot of ln (*A* *t* − *A*_*∞*_) versus time. The value of ∆ that corresponds at time *t* = 1 × 10^3^ s is shown. Conditions: [2,3-DHBA]_0_ = 0.039 M, [Cr(III)]_0 _ = 0.05 *Μ*, *d* = 1 cm cell, and *T* = 289 K.

The plots of ln (*A*_*t*_ − *A*_*∞*_) where *A*_*t*_ and *A*_*∞*_ are absorbances at time *t* and after the completion of the corresponding reaction step against time were found to be non-linear (Figure 4); they have curvature at short reaction times and have a constant slope at larger reaction times. 

**Figure 5:**
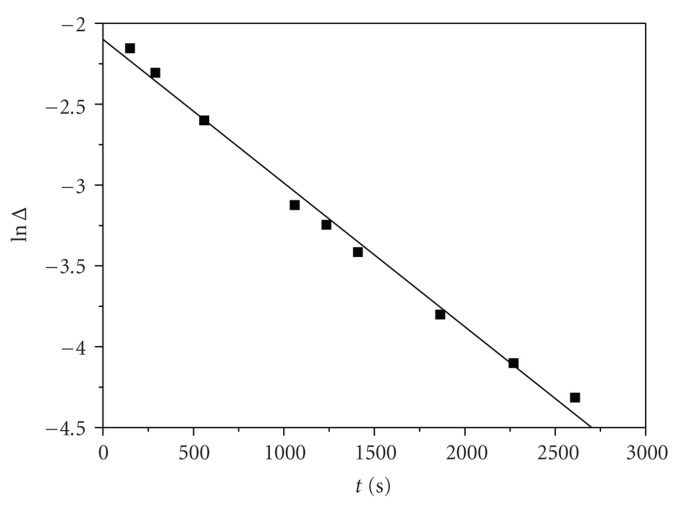
A typical plot of ln Δ versus time. Conditions: [2,3-DHBA]_0_ = 0.039 M, [Cr(III)]_0_ = 0.05 *Μ*, *d* = 1 cm cell, and *T* = 289 K.

Contribution of the uncomplexed Cr(III) species in the absorbance values, mainly due to the excess of Cr(III), does not interfere in the graphs (Figures 4 and 5) because it is included in both *A*_*t*_ and *A*_*∞*_ and is thus eliminated due to the subtraction of *A*_*∞*_ from *A*_*t*_. The recorded values of *A*_*∞*_ were very close to the true values which were obtained by plotting *A* = *f*(*t*); in this way confirmation of the completion of the certain step of the reaction was possible.

The rate constants were calculated according to methods found in the literature [7, 8], assuming two consecutive first-order (or pseudo-first-order) steps:

(2) $$\text{A}\overset{k_{2}}{\to }\text{B}\overset{k_{3}}{\to }\text{C}.$$

The above reaction sequence admits of two mathematical solutions, and the sets of the rate constants are such that the fast and slow kinetic steps are interchanged [7, 9, 10]. 

The *k*_3(obs)_ values were obtained from the slope (−*k*_3_) of the linear second part (long-time part) of the ln (*A*_*t*_ − *A*_*∞*_) = *f*(*t*) plot.

The *k*_2(obs)_ values for A → B step were evaluated by the method of Weyh and Hamm [8] using the rate equation

(3) $$A_{t}-A_{\infty }=a_{2}\cdot e^{-k_{2(\text{obs})}t}+a_{3}\cdot e^{-k_{3(\text{obs})}t}.$$

Values of *α*_2_ and *α*_3_ are dependent upon the rate constants and the extinction coefficients. At various times *t*, Δ = *A*_*t*_ − *A*_*∞*_ − *a*_3_ · *e* ^−*k*_3(obs)_*t*^ = *a*_2_ · *e* ^−*k*_2(obs)_*t*^ (Figure 4). Hence, ln Δ = constant − *k*_2(obs)_ *t* and the *k*_2(obs)_ values were found from the slope of the plots of ln Δ versus *t* for small values of reaction time. A typical plot appears in Figure 5.

At times longer than three to four half-lives of the B → C step, a reaction involving oxidation of the ligand takes place, resulting in anomalous further absorbance changes. This reaction was not studied.

The above assumptions for the existence of two consecutive steps for the reaction under study and the calculation of *k*_2(obs)_ and *k*_3(obs)_ values fit with all the experimental data, at all temperatures. Table 1 gives the *k*_2(obs)_ and *k*_3(obs)_ values for this reaction of 2,3-DHBA with Cr(III) at various temperatures (289 K, 293 K, 297 K, 304 K, and 310 K).

**Figure 6:**
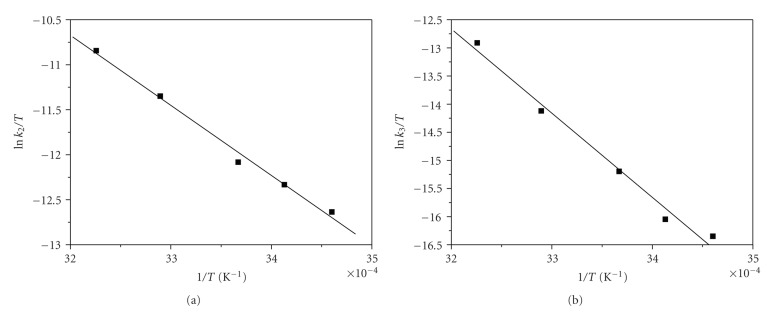
(a) Eyring plot for step 2. (b) Eyring plot for step 3.

The activation parameters Δ*H*_2(obs)_^≠^, Δ*S*_2(obs)_^≠^, and Δ*H*_3(obs)_^≠^, Δ*S*_3(obs)_^≠^ corresponding to *k*_2(obs)_ and *k*_3(obs)_, respectively, were calculated from the linear Eyring plots (Figures 6(a) and 6(b)) and are presented in Table 2. 

### 2.3. pH Measurements

**Figure 7:**
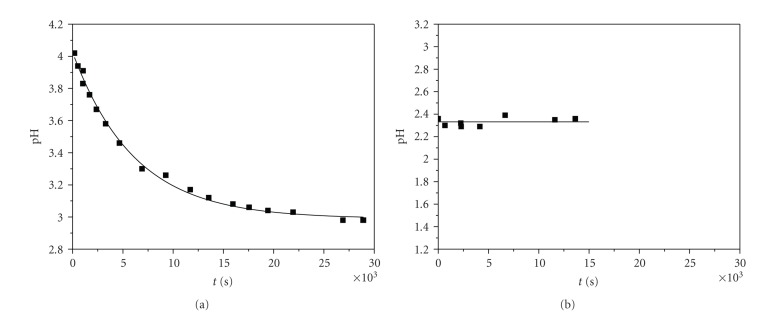
(a) pH = *f*(*t*) for the reaction between 2,3-DHBA and Cr(III) in aqueous solutions. Conditions: [2,3-DHBA]_0_ = 0.01 M, [Cr(III)]_0_ = 0.10 M, [KOH]_0_ = 0.1 M (stock solution added in the mixture), and *T* = 298 K. (b) pH = *f*(*t*) of a blank sample. Conditions [Cr(III)]_0_ = 0.1 M, and *T* = 298 K.

Measurements of the pH values of various reaction mixtures versus time at constant temperatures were recorded using a SANXIN PHS-3D pH model pH meter. These values were plotted against time giving pH = *f*(*t*), that is, −log [*H* ^+^] = *f*(*t*). A typical graph is shown in Figure 7(a). A pH = *f*(*t*) graph of a blank sample, containing only Cr(III) solution is presented in Figure 7(b), for comparison.

### 2.4. Isolation in the Solid Form

The isolation of the final product of the reaction (complex C) in the solid form from Cr(III)/2,3-DHBA mixtures was achieved by addition of quantities of KOH in reaction mixtures of various stoichiometries. The elemental analyses show that only one ligand molecule enters the coordination sphere of the metal and they were conducted for C, H, and N and gave C = 10.62%, H = 3.66%, and N = 1.38%.

## 3. Results and Discussion

**Scheme 1:**
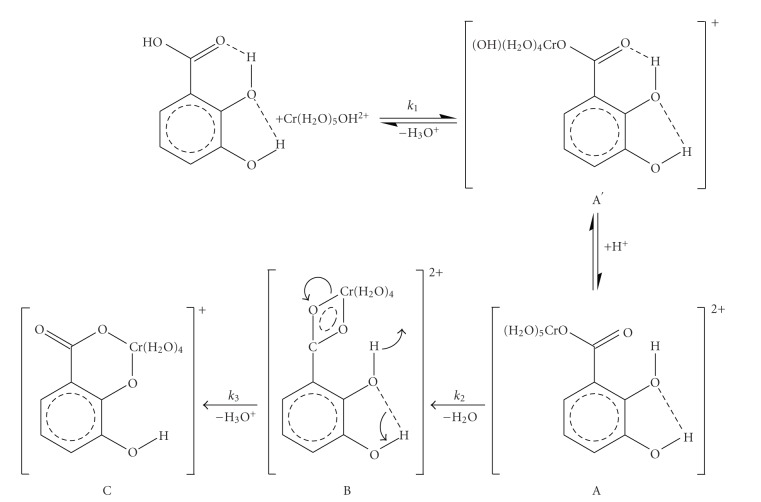
A possible mechanism of the reaction between chromium(III) and 2,3-dihydroxybenzoic acid in weak acidic aqueous solutions.

The form of the metal ion that reacts with the ligand 2,3-DHBA is [Cr(H_2_O)_5_OH]^2+^ since it is well known that the hydroxy complex [Cr(H_2_O)_5_(OH)]^2+^ is highly more reactive than [Cr(H_2_O)_6_]^3+^. The *K*_*a*_ for the reaction

(4) $$\text{Cr}{\left({\text{H}}_{2}\text{O}\right)}_{6}^{3+}⇌\text{Cr}{\left({\text{H}}_{2}\text{O}\right)}_{5}{\left(\text{OH}\right)}^{2+}+{\text{H}}^{+}$$

is about 10^−4^ [11, 12]. At pH lower than 4 the chromium(III) complex exists mainly in the hexa-aqua monomeric form. Its spectrum shows maxima at 575 nm and 410 nm (at the visible region). An amount of [Cr(H_2_O)_5_(OH)]^2+^ is though always present under the above conditions. In the experiments reported here that are conducted over the pH range 3-4 the reaction should be considered as taking place first with [Cr(H_2_O)_5_(OH)]^2+^ rather than with [Cr(H_2_O)_6_]^3+^, since a fast first step is taking place and it is very well known that Cr(H_2_O)_6_^3+^ is very substitution inert. In the ligand molecule there are two intramolecular hydrogen bonds, one between adjacent hydroxyl groups and the other between the hydroxyl group and the carbonyl oxygen atom [13] (Scheme 1).

The violet [Cr(H_2_O)_6_]^3+^ (Figure 2(a)) and the light brown solution of the ligand (Figure 2(b)) on mixing give a violet-colored solution due to the excess of [Cr(H_2_O)_6_]^3+^ since the final UV-Vis spectrum resembles that of [Cr(H_2_O)_6_]^3+^.

The formation and subsequent transformation (substitution) kinetics suggest that a first complex A is formed (Scheme 1). In the kinetic experiments we assume that we start with complex A. This implies that the subsequent two slow steps, *k*_2_ and *k*_3_ (which were studied), did not contribute to the formation of complex A (first step) at the region of temperatures studied (16–37°C).

**Figure 8:**
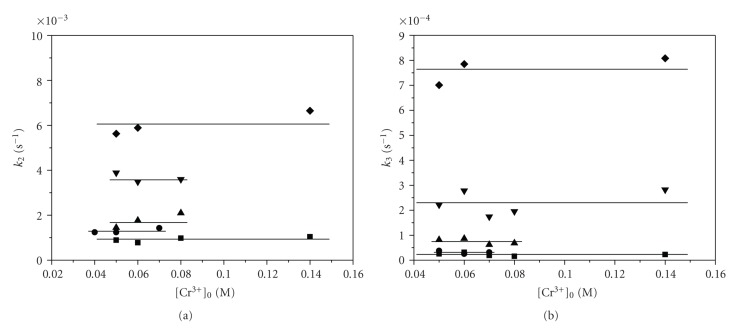
(a): Dependence of *k*_2(obs)_ on Cr(III) concentrations at various temperatures for the reactions $\text{A}\overset{k_{2}}{\to }\text{B}\overset{k_{3}}{\to }\text{C}.$ –■– 289 K, –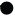
– 293 K, –▴– 297 K, –▾– 304 K, –♦– 310 K. (b): Dependence of *k*_3(obs)_ on Cr(III) concentrations at various temperatures for the reactions $\text{A}\overset{k_{2}}{\to }\text{B}\overset{k_{3}}{\to }\text{C}.$ –■– 289 K, –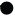
– 293 K, –▴– 297 K, –▾– 304 K, –♦– 310 K.

The ln (*A*_*t*_ − *A*_*∞*_) versus time plots are indicative of a complex reaction, actually a consecutive two first-order steps series of reaction (according to the analysis described in the experimental part). The two slow consecutive steps were found to be nondependent on chromium(III) concentration (Figures 8(a) and 8(b)), implying that a first fast complexation step took already place. Thus, at least three steps *k*_1_, *k*_2_, and *k*_3_ are taking place (Scheme 1).

The first step (*k*_1_) that is taking place with the [Cr(H_2_O)_5_OH]^2+^ reacting species proves the suggested reaction through the hydroxy form of chromium(III) and not through the hexa-aqua form, [Cr(H_2_O)_6_]^3+^, since the substitution rate constants of the latter species would be very small.

The carboxylic and hydroxyl groups of the ligand (2,3-DHBA) are blocked (protonated, or hydrogen-bonded) and so the attacks by chromium(III) can take place only by removing protons, a fact that is measured by the pH decrease of the solution (Figure 7(a)). 

 A possible mechanism consistent with all experimental data is shown in Scheme 1. The attack of [Cr(H_2_O)_5_(OH)]^2+^ at the hydrogen-bonded carboxylic group of 2,3-DHBA leads to complex A; complexation results in destruction of the hydrogen bonding; the consequence of the [Cr(H_2_O)_5_(OH)]^2+^ usage in complexation is shift of the equilibrium of (4) to the right.

In conjugate base mechanisms the conjugate base, being present as a small fraction of the total, reacts and then takes a proton as it would naturally do. So, rapid protonation equilibrium follows the first formed species (A′) favoring the formation of the aqua species (complex A). 

The reaction of [Cr(H_2_O)_5_(OH)]^2+^ with the hydrogen-bonded ligand results in substitution of a water molecule in the Cr(III) coordination sphere, by the carboxylic group, with the water molecule being the one that is located trans to the OH group of the species [Cr(H_2_O)_5_(OH)]^2+^ and is labile. For step 1 (*k*_1_) an *I*_*d*_ mechanism is suggested since the conjugate base [Cr(H_2_O)_5_(OH)]^2+^ reacts according to such a mechanism [14]. This is because of the strong labilizing effect, which is induced by the coordinated OH^−^, presumably, on the trans H_2_O molecule. This leads to a 10^2^–10^3^-fold enhanced reaction rate for the hydroxy-aqua over the hexaaqua ion [14].

**Scheme 2:**
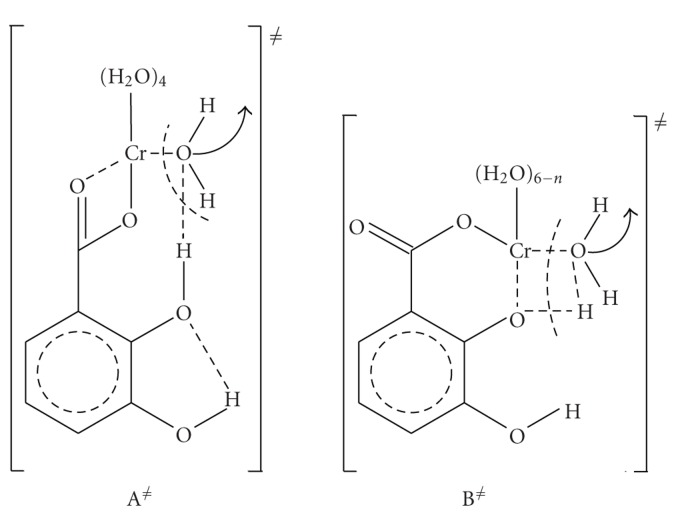
Activated complexes A^≠^ and B^≠^.

Complex A reacts in two consecutive steps (*k*_2_, *k*_3_) to give B and C. The activation parameters deduced from the temperature-dependence experiments are used for proposing structures of the activated complexes (Scheme 2) and for proposing the mechanisms that are taking place (Scheme 1). 

The spectra of Figure 3 correspond to the reaction mixture at various times after mixing at 296 K, and the mixture contains the species A, B, and C (Scheme 1). 

The *k*_2(obs)_ and *k*_3(obs)_ dependence on chromium(III) concentration (Figures 8(a) and 8(b)) was studied at the temperature range 16–37°C in order (a) to find if a second or third chromium(III) ion is reacting with the already formed complex A and (b) to be able to calculate the activation parameters Δ*H* ^≠^ and Δ*S* ^≠^, for the two steps. The studies show that (a) the two steps are independent on Cr(III) concentration meaning that the transformations taking place in the two steps are taking place within the already formed first complex A, that is, chelation to form complex B and then breaking of one Cr–O bond and formation of another Cr–O bond in order to form a new chelate, that bears less tension, that is, it is more stable. The four-membered ring is transformed at step 3 to a six-membered ring. (b) The calculations revealed that, going from complex A to complex B, Δ*S* ^≠^ < 0, that is, the transition state is more organized than the reactants, i.e. complex A^≠^ is having chelate rings, that is, less freedom than complex A. From complex B to complex C,  Δ*S* ^≠^ > 0, that is, there is increase in freedom and so the complex B^≠^ is less organized than complex B having a six-membered ring instead of a four-membered ring and not having a hydrogen bond that exists in complex B. 

For step 2 (*k*_2_) the negative value of Δ*S* ^≠^, the independence of *k*_2_ on Cr(III) concentrations and the increase in absorbance, that is, of the extinction coefficients, led to the assignment of the observed transformations as *associatively activated substitution *of water molecules from the Cr(III) coordination sphere. Associative mechanism has been supported to operate in reactions of Cr(III) [15–17]. For step 3 (*k*_3_) a positive Δ*S* ^≠^ is observed indicating a less organized transition state than the reactants (complex B). This suggests more degrees of freedom in the corresponding transition state. Actually the suggestion of a six-membered ring (complex C) instead of a four-membered ring (step 2, complex B) is having less tension. Also the destruction of the hydrogen bond gives more freedom and thus less organization in the molecule explaining the positive entropy of activation of step 3. Since associative mechanism has been supported to operate in reactions of Cr(III), the transformations that take place at the Cr(III) center are associatively activated but the overall rearrangements that take place at the activated stage lead to overall positive value of the entropy of activation. 

By isolating the Cr(III)/2,3-DHBA final complex (complex C) in the solid form, elemental analyses were conducted and are in agreement with a 1 : 1 Cr(III)/2,3-DHBA ratio of complex C. An empirical formula for complex C is suggested according to the elemental analysis data: [2,3-DHBA-_3H_ · Cr(H_2_O)_4_K · KNO_3_ · 6H_2_O · 4KOH] for which the calculated percentages are C = 11.2%, H = 3.61%, and N = 1.87%. The experimental values are C = 10.62%, H = 3.66%, and N = 1.38%. The catecholic mode of binding was found to operate in the coordination complexes of 3,4-dihydroxyphenylpropionic acid (dihydrocaffeic acid), 3,4-dihydroxyphenylpropenoic acid (caffeic acid), and 3,4-dihydroxybenzoic acid (3,4-DHBA) with Cr(III) [18–20]. This type of binding was also reported for complexes of dihydrocaffeic, caffeic, and ferulic acids with Co(II), Ni(II), Cu(II), Fe(III), Mn(II), Mn(III), V(V), V(IV,V), and Zn(II) [21–25]. In the case of the reaction of 2,3-dihydroxybenzoic acid with chromium(III) the strong hydrogen bonds that exist intra- and intermolecularly cause the deviation from the above mode of binding. Though the substitution mechanisms at the chromium(III) center are associative (Δ*S* ^≠^ < 0, step 2, step 3), the total change in the entropy of activation in the final step (step 3) is finally positive due to the destruction of the remaining hydrogen bond at the transition state of step 3, along with the destruction of a four-membered ring and the formation of a six-membered ring. This positive value of Δ*S*_3_^≠^ led us to suggest the mechanism that is shown in Scheme 1, not proposing a final(catecholic)chelation step as in the previous cases [18–20] that were studied by us.

## 4. Conclusions

The reaction between Cr(III) and 2,3-Dihydroxy-benzoic acid in weak acidic aqueous solutions was investigated, and the experimental results are consistent with a three-step mechanism in which the initial attack (step 1, substitution of water molecules from the coordination sphere of Cr(III) by the ligand through complexation) takes place between the acid molecule and the [Cr(H_2_O)_5_OH]^2+^complex following an *I*_*d*_ mechanism. The carboxylate bound Cr(III), complex A, is followed by two consecutive nonchromium(III)-dependent steps (step 2 and step 3). These two steps are assigned as chelation and isomerisation steps, supported to be associatively activated at the Cr(III) center. The reactions are followed by a pH decrease because proton release is taking place during the overall mechanism. The negative value of the entropy of activation of step 2, the positive value of the entropy of activation of step 3, the independence on Cr(III) concentrations, the increase of the extinction coefficients, the pH decrease due to the release of protons upon the various transformations and the 1 : 1 stoichiometry of the final complex C led to the proposed mechanism in Scheme 1.

## Figures and Tables

**Table 1:** Values of *k*_2(*obs*)_ and *k*_3(*obs*)_ at various temperatures.

*k*_2(obs)_× 10^3^ (s^−1^)	*k*_3(obs)_× 10^5^ (s^−1^)	*T* (K)
0.94	2.29	289
1.29	3.14	293
1.68	7.46	297
3.58	23.02	304
6.05	76.47	310

**Table 2:** Activation parameters for steps $\text{A}\overset{k_{2}}{\to }\text{B}$ and $\text{B}\overset{k_{3}}{\to }\text{C}.$

Δ*H*_2(obs)_^≠^ (kJ mol^−1^)	Δ*S*_2(obs)_^≠^ (JK^−1^ mol^−1^)	Δ*H*_3(obs)_^≠^ (kJ mol^−1^)	Δ*S*_3(obs)_^≠^ (JK^−1^ mol^−1^)
61.2 ± 3.1	−91.1 ± 11.0	124.5 ± 8.7	95.1 ± 29.0
